# The Effect of Nanoparticles Against Streptococcus mutans in the Orthodontic Primer Used for Aligner Attachment: An In Vitro Study

**DOI:** 10.7759/cureus.68359

**Published:** 2024-09-01

**Authors:** Ram Mundada, Saurabh B Tanpure, Sagar Mapare, Arjun Karra, Vijay Yannawar, Rizwan Gilani

**Affiliations:** 1 Department of Orthodontics and Dentofacial Orthopedics, Dr. Hedgewar Smruti Rugna Seva Mandal's Dental College and Hospital, Hingoli, IND; 2 Department of Orthodontics and Dentofacial Orthopedics, Sharad Pawar Dental College, Datta Meghe Institute of Higher Education and Research, Wardha, IND

**Keywords:** attatchment, aligners, orthodontic primer, s.mutans, nano particles

## Abstract

Objective

This study investigates the antimicrobial properties of silver (1%) and chitosan (1%) nanoparticles against Streptococcus mutans (S. mutans) when added to an orthodontic primer used for aligner attachments. While aligner treatments are becoming increasingly popular for their aesthetics and convenience, their attachments can create retention sites for bacteria, potentially leading to white spot lesions (WSLs). This in vitro study aims to address this issue by enhancing the antimicrobial efficacy of aligner primers.

Methodology

Thirty freshly extracted teeth were classified into the following three groups: Group A with the standard primer, Group B with chitosan nanoparticles mixed in the primer, and Group C with silver nanoparticles mixed in the primer. The samples were incubated with S. mutans and bacterial colonies were counted at 12, 24, 48, and 72 hours.

Results

The results showed a significant reduction in colony-forming units (CFUs) in the groups with nanoparticles compared to the control group, with silver nanoparticles exhibiting a higher antimicrobial effect than chitosan.

Conclusions

This study suggests that incorporating silver nanoparticles into orthodontic primers can effectively reduce bacterial growth, potentially improving oral hygiene and reducing the risk of WSLs in patients undergoing aligner treatment.

## Introduction

Treatment using aligners has gained popularity among adult and adolescent patients due to the demand for better aesthetics and health [1]. When compared to fixed appliances, aligners have better aesthetics, less chair time, removability, and reduced visits [2]. Furthermore, patients undergoing aligner treatment reportedly experience lower pain levels during the initial days of treatment [3]. Aligners bring ease in maintaining oral hygiene, reducing enamel demineralization, and the formation of biofilms in comparison with fixed appliances. However, a study showed there was no significant difference between the oral microbiome of patients treated with fixed appliances and those with clear aligners [4]. Acquired pellicle and biofilm accumulate over the tooth surface and gingiva as these aligners are worn for more than 22 hours per day [5]. Patients with clear aligner treatment are more prone to develop shallow but large white spot lesions (WSLs) than those with fixed orthodontic treatment [6].

Attachments in clear aligners are small tooth-colored configurations used in almost every clear aligner treatment. These attachments improve the contact between the aligner and the teeth and ensure efficient tooth movements and better retention of the aligner [7]. However, due to their irregular shapes, the attachments create retention sites on tooth surfaces, making it easier for bacteria and food particles to accumulate, which often leads to poor oral hygiene, and the risks of WSLs increase [8]. After the treatment, attachments are removed for aesthetic reasons. This can be challenging as the attachment resin is similar in color to the enamel. Inadequate removal can cause discoloration and bacterial buildup, while excessive removal can damage the enamel. Therefore, a new clear aligner resin and primer attachment needs to be developed, which can be differentiated from enamel and has antibacterial properties [9].

The effect of nanoparticles on microbial loads has been extensively studied. These antimicrobial metal nanoparticles are silver oxides, titanium oxides, and chitosan; in the present study, silver nanoparticles and chitosan effects are studied. The silver nanoparticles 1-10 nm in size did show a very high microbicidal property. Silver nanoparticles added in the resin release silver ions that act against oral streptococci. Another antibacterial agent analyzed in the study is chitosan, which is formed by the deacetylation of chitin and is a naturally acquired polysaccharide [10]. Chitosan acts by inhibiting the enzyme action by the formation of acidic polymers, which are similar to teichoic acid, and substituting the lipopolysaccharides and metal ions.

The study aims to evaluate the antimicrobial properties of silver (1%) and chitosan (1%) nanoparticles against Streptococcus mutans (S. mutans) when added to an orthodontic primer used for aligner attachment and to compare the cidal effect of the two.

## Materials and methods

Sample size estimation

Sample size (n) estimation was done by “comparing two means” formula by using open Epi (v.3.0) software:

n = (σ12 + σ22/k) (Z1-α/2+Z1-β)2

 Δ2

Where, s 1 = Group 1 standard deviation, s 2 = Group 2 standard deviation, and D = difference between group means (Table 1).

**Table 1:** Difference between group means

	Group 1	Group 2	Difference
Mean	51.25	132.08	80.83
Standard deviation	12.40	73.52	
Variance	153.76	5405.19	

S. mutans was extracted from a salivary sample with the help of a polymerase chain reaction (PCR) machine and cultured as per guidelines.

Preparation of beef heart infusion (BHI)

Beef heart (infusion from 250g), 5 g/L; calf brains (infusion from 200g), 12.5 g/L; disodium hydrogen phosphate, 2.5 g/L; D(+)-glucose, 2 g/L; peptone, 10 g/L; sodium chloride, 5 g/L. Bacterial suspension and teeth samples were mixed in the test tube. Samples were vortex incubated for 24 hrs at 37 °C; 0.1 ml of the above suspension was subcultured for the following time intervals: 12, 24, 48, and 72 hours. After respective time intervals, samples were plated on the nutrient agar plate and 28.0 grams were suspended in 1000 mL of distilled water. It was heated until boiling to completely dissolve the medium. Sterilization by autoclaving was done at 15 lbs pressure (121 °C) for 15 minutes and cooled to 45-50 °C. We can also enrich the medium with 5-10% blood or biological fluids. It was mixed well and poured into a sterile Petri dish and incubated for 24 hrs at 37 °C. Colonies were counted by colony counters.

Statistical analysis 

Statistical analysis was done using SPSS Statistics version 21.0 (IBM Corp., Armonk, NY). Descriptive statistics were calculated for all groups based on different parameters. Repeated measures one-way Analysis of Variance (ANOVA), followed by Tukey’s post hoc test, was used to assess significant differences between the three groups. A p-value less than 0.05 was considered statistically significant at the 95% confidence level.

## Results

**Figure 1:**
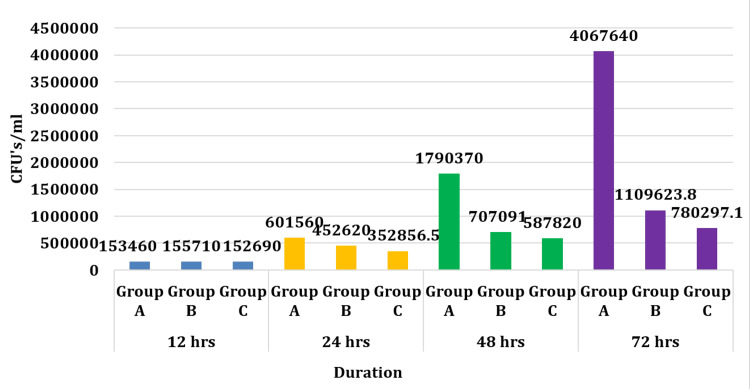
Bar graph showing CFUs in each group at different time intervals CFU: colony-forming unit

**Figure 2:**
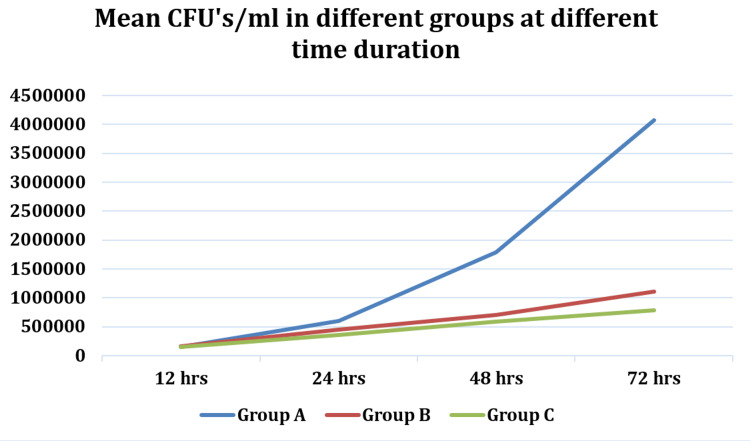
Line diagram showing CFUs in each group at different time intervals CFU: colony-forming unit

In our study, the intergroup comparison of colony-forming unit (CFU)/ml between different groups at 12 hours, 24 hours, 48 hours, and 72 hours was calculated using repeated measures ANOVA, followed by Tukey’s post hoc test (Tables 2-4; Figures 1-2).

**Table 2:** Descriptive statistics of CFUs/ml in different groups at different time intervals CFU: colony-forming unit

Time duration	Groups	N	Mean (CFUs/ml)	Std. deviation
12 hrs	Group A	10	153460	10662.93
	Group B	10	155710	10948.00
	Group C	10	152690	10819.06
24 hrs	Group A	10	601560	9497.62
	Group B	10	452620	13265.47
	Group C	10	352856.5	9557.61
48 hrs	Group A	10	1790370	10370.47
	Group B	10	707091	4755.38
	Group C	10	587820	7201.04
72 hrs	Group A	10	4067640	22147.64
	Group B	10	1109623.8	11200.20
	Group C	10	780297.1	427.04

**Table 3:** Intergroup comparison of CFUs/ml between different groups at 12, 24, 48, and 72 hrs CFU: colony-forming unit

Comparison groups		Mean difference (I-J)	P-value	95% confidence interval	
				Lower bound	Upper bound
Group A	Group B	1046996.3000	0.005	1039615.0395	1054377.5605
	Group C	1184841.6000	0.000	1177460.3395	1192222.8605
Group B	Group A	-1046996.3000	0.005	-1054377.5605	-1039615.0395
	Group C	137845.3000	0.000	130464.0395	145226.5605
Group C	Group A	-1184841.6000	0.000	-1192222.8605	-1177460.3395
	Group B	-137845.3000	0.000	-145226.5605	-130464.0395

**Table 4:** Comparison of CFUs/ml between different time intervals (12, 24, 48, and 72 hrs) across different groups CFU: colony-forming unit

Time (I)	Time (J)	Mean difference (I-J)	P-value	95% confidence interval	
				Lower bound	Upper bound
12 hrs	24 hrs	-315058.833	0.001	-322774.178	-307343.489
	48 hrs	-874473.667	0.000	-881452.599	-867494.734
	72 hrs	-1831900.300	0.003	-1840656.745	-1823143.855
24 hrs	12 hrs	315058.833	0.001	307343.489	322774.178
	48 hrs	-559414.833	0.000	-565838.733	-552990.933
	72 hrs	-1516841.467	0.000	-1524664.157	-1509018.777
48 hrs	12 hrs	874473.667	0.000	867494.734	881452.599
	24 hrs	559414.833	0.000	552990.933	565838.733
	72 hrs	-957426.633	0.000	-965406.284	-949446.983
72 hrs	12 hrs	1831900.300	0.003	1823143.855	1840656.745
	24 hrs	1516841.467	0.000	1509018.777	1524664.157
	48 hrs	957426.633	0.000	949446.983	965406.284

This comparison revealed statistically significant differences (p<0.05) between all groups at each time interval.

The comparison of CFU/ml between different time intervals (12 hours, 24 hours, 48 hours, and 72 hours) across different groups was performed using repeated measures ANOVA. This analysis showed statistically significant differences (p<0.05) between all-time intervals across all groups.

## Discussion

The ubiquity of WSLs is increasing with the increased usage of aligners for orthodontic treatment. Wearing clear aligners for an extended period affects the inflow of saliva, thus impairing the beneficial effects of saliva on tooth surfaces [6]. S. mutans was first isolated by J. Clarke in 1924 and has gained widespread attention as an important etiology agent in dental caries since then [11]. S. mutans secrete glycosyltransferases (GTFs) that get absorbed on bacterial surfaces and enamel pellicles. In the presence of sucrose, GTFs catabolize sugar and produce glucans that help build up the extracellular polymeric matrix (EPS). This EPS matrix acts as a mediating scaffold to which bacteria and tooth enamel adhere. Continuous sugar intake by the host leads to the growth of this acidogenic bacteria, i.e., S. mutans, thereby lowering the PH and initiating demineralization and carious process [12]. Thus, as an alternative approach to reduce the bulk of bacterial colonies over the aligner attachment, various antimicrobial agents are being either added in primer or composites.

Silver nanoparticles act against bacteria by disrupting the cell wall and cytoplasmic membrane or by denaturation ribosomes, inhibiting protein formation, or by interruption of ATP production. Another way silver nanoparticles act against bacteria is by interfering with DNA replication [13]. Chitosan also acts against bacteria by districting bacterial cell walls, interfering with DNA replication, and chelation of nutrients [14]. Felip et al. [15] showed that an increased percentage of silver nanoparticles decreases the S. mutans colonies significantly. Juan et al. [16] concluded that silver nanoparticles at lower concentrations than gold and zinc have a more antimicrobial effect. Botan et al. [17] concluded in their study that adding chitosan in the clear aligner resin at 3% and 5% significantly decreased CFUs by 31% and 70% respectively. Thus, in our study, we took 1% silver nanoparticle because it would have a higher bactericidal effect even at such a low concentration. Jesus et al. [18] concluded in their study that silver nanoparticles can also be added to orthodontic adhesives without altering the properties of the adhesive while maintaining improved bactericidal activity [19].

In the present study, bacterial colony growth was studied in three different groups. The two groups contained chitosan and silver nanoparticles separately and the third group was kept as a control with no nanoparticle added in the primer. The chitosan and silver were added in the primer separately and coated on the tooth enamel surface before the bonding clear attachments. The teeth samples then were transferred to a pathological lab to count the CFUs. During the first 12-24 hrs, there was no significant growth in all three groups. In between 48 to 72 hrs, there was a significant rise in CFUs in the control group, whereas, in the chitosan and silver groups, the CFUs were low, suggesting a good antibacterial property of these two nanoparticles against S. mutans. A comparison of the cidal effect between the chitosan and silver nanoparticle groups showed that silver nanoparticles are more effective in controlling S. mutans count than the 1% chitosan group (Tables 2-4; Figures 1-2).

Since previous studies have shown that the long-term effect of nanoparticles has non-significant results [20], we studied the effect of nanoparticles in the short term. Also, till now, these two nanoparticles were not used in aligner attachment, which also prompted us to conduct this study. Thus, when each of the nanoparticles, either silver or chitosan, was added to the primer at the same 1% concentration, the silver nanoparticle showed more antibacterial effect than the chitosan group, which corresponds to the findings of Felip et al. [16] (more than 0.33% have a bactericidal effect) and Botan et al. [17] who reported that the bactericidal effect was less with 3% chitosan.

This study has a few limitations, primarily its in vitro design, which may not fully replicate the oral environment. Additionally, only short-term effects were evaluated. Future studies should preferably involve in vivo trials to assess long-term efficacy, potential bacterial resistance, and the optimal nanoparticle concentration for safe and effective use in orthodontic primers.

## Conclusions

Based on our findings, incorporating 1% silver nanoparticle and 1% chitosan nanoparticles significantly increases the antimicrobial activity of the orthodontic primer, with silver nanoparticles being more effective. The highest antimicrobial efficacy was seen at 72 hrs. Hence, the addition of silver nanoparticles to the primer can be a potential alternative for improving treatment outcomes.
